# Supplementary material for: MARCH8 inhibits influenza A virus infection by targeting viral M2 protein for ubiquitination-dependent degradation in lysosomes
Source: Nat Commun. 2021 Jul 20;12:4427. doi: 10.1038/s41467-021-24724-2 (PMC8292393; doi:10.1038/s41467-021-24724-2)
Supplement: Supplementary file 1 — Supplementary information [file 41467_2021_24724_MOESM1_ESM.pdf]

Supplementary information

MARCH8 inhibits influenza A virus infection by targeting viral M2 protein for ubiquitination-dependent degradation in lysosomes

Xiaoman Liu, Fengwen Xu, Lili Ren, Fei Zhao, Yu Huang, Liang Wei, Yingying Wang, Conghui Wang, Zhangling Fan, Shan Mei, Jingdong Song, Zhendong Zhao, Shan Cen, Chen Liang, Jianwei Wang, Fei Guo

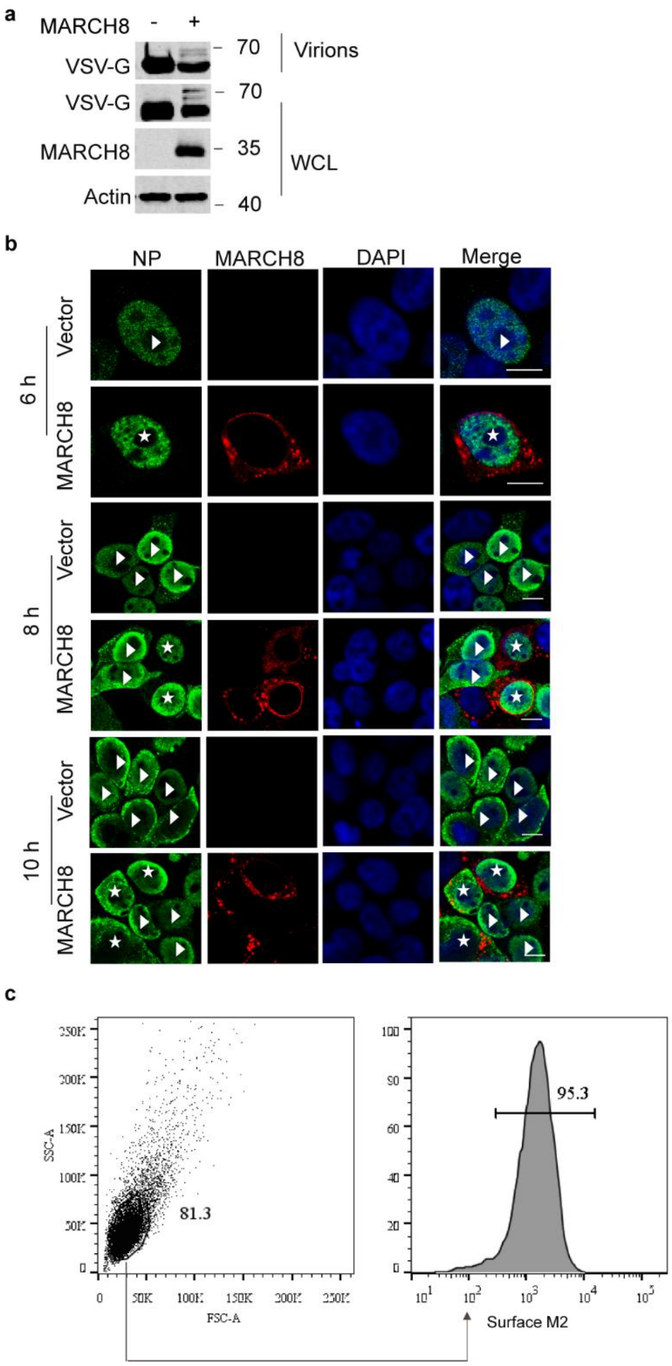

Supplementary Figure 1. VSV-G expression and IAV NP trafficking in MARCH8 overexpression cells. a. Production of Lenti-GFP reporter viruses pseudotyped with VSV-G in HEK293T cells without or with MARCH8 plasmid DNA. VSV G protein associated with cell lysates and the released virus particles was detected by Western blot. **b**. Immunostaining of NP and MARCH8. HEK293T cells transfected with either a control vector or the MARCH8 plasmid DNA were infected with WSN virus (MOI = 2). At the indicated time points, cells were subject to staining

with NP and MARCH8 antibodies. NP localization in control cells is indicated with arrows and in MARCH8 expression cells is indicated with stars. **c.** Gating strategy for detection of Surface M2 within live cell populations.

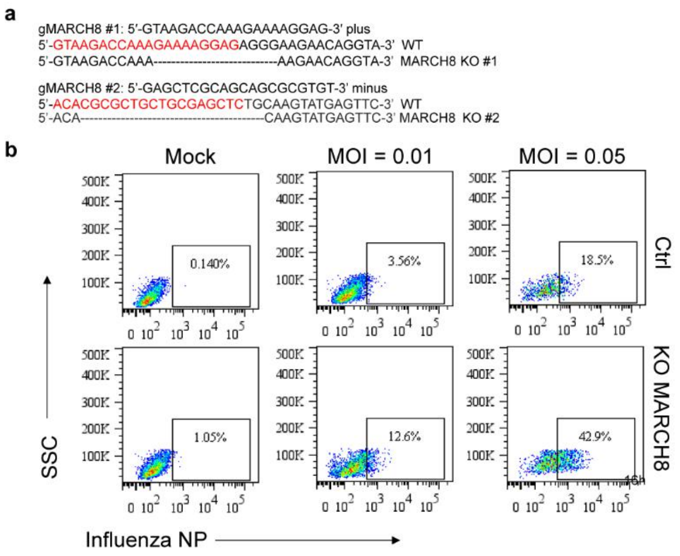

Supplementary Figure 2. Knockout of MARCH8 promotes IAV replication. a. Knockout of the *MARCH8* gene was determined by Sanger sequencing. **b**. Ctrl and KO MARCH8 A549 cells were infected with WSN virus at the indicated MOI. At 24 h post-infection, viral NP protein expression were measured by flow cytometry.

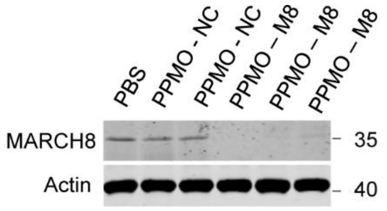

Supplementary Figure 3. PPMO efficiently knockdown MARCH8 in mice lung. Six-week old female C57 B/6 mice were administered PBS, control PPMO (PPMO-NC) or PPMO targeting mouse MARCH8 (PPMO-M8) intranasally for 2 consecutive days. MARCH8 protein levels were determined in lungs on day 0 without IAV infection.

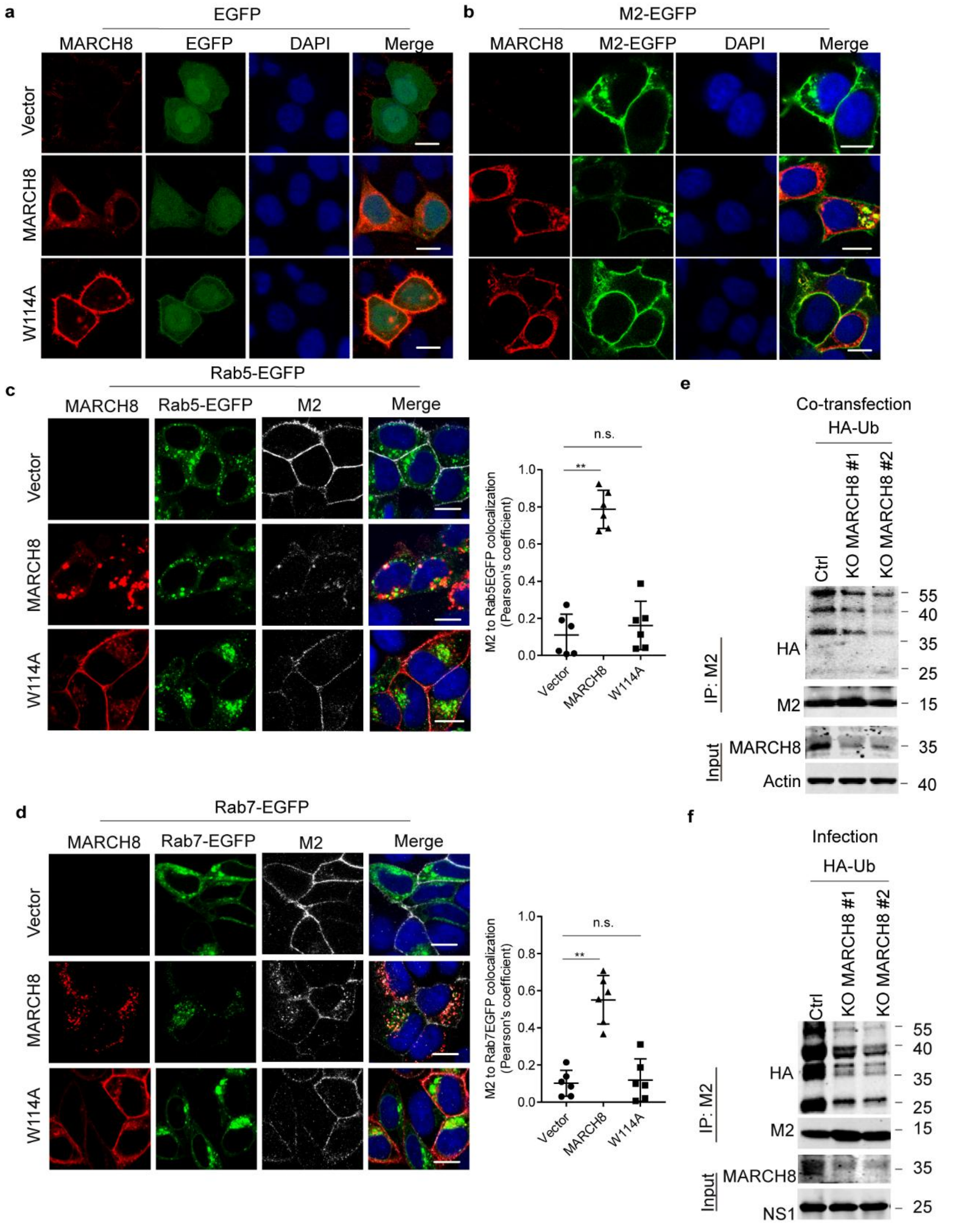

Supplementary Figure 4. MARCH8 targets M2 for degradation in lysosomes. a and **b.** HeLa cells transfected with plasmids encoding EGFP (**a**) or EGFP fused M2 (**b**) plus Vector, MARCH8 or W114A were permeabilized and stained with antibody against MARCH8. Scale bars represent 10 μm. **c** and **d.** HeLa cells were transfected with EGFP fused Rab5 (**c**) or Rab7 (**d**) plus M2, and vector, MARCH8 or W114A.

Cells were permeabilized and co-stained with antibodies against M2 and MARCH8. Scale bars represent 10 μm. Pearson's correlation coefficient analysis was based on multiple sight fields each group (n = 6). **, P<0.001; n.s., nonsignificant, unpaired two-tailed Student t-test. **e** and **f.** Control (Ctrl) or MARCH8-knockout (MARCH8 KO #1, #2) HEK293T cells were transfected with HA-Ub and M2 DNA (**e**). Control (Ctrl) or MARCH8-knockout (MARCH8 KO #1, #2) HEK293T cells transfected with HA-Ub were infected with WSN virus (MOI = 2) for 12 h (**f**). Whole-cell lysates were subjected to IP with anti-M2 antibody, and the IP and input were analyzed by western blotting with antibodies against the indicated targets.

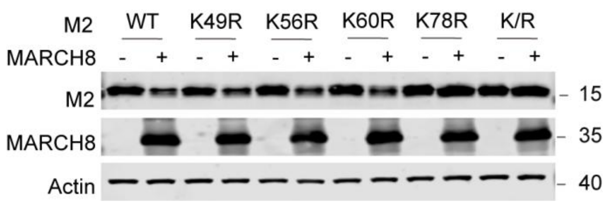

Supplementary Figure 5. Effect of MARCH8 on expression of M2 mutants. HEK293T cells were transfected with M2 mutants and vector or MARCH8. Total M2 was determined by western blot.

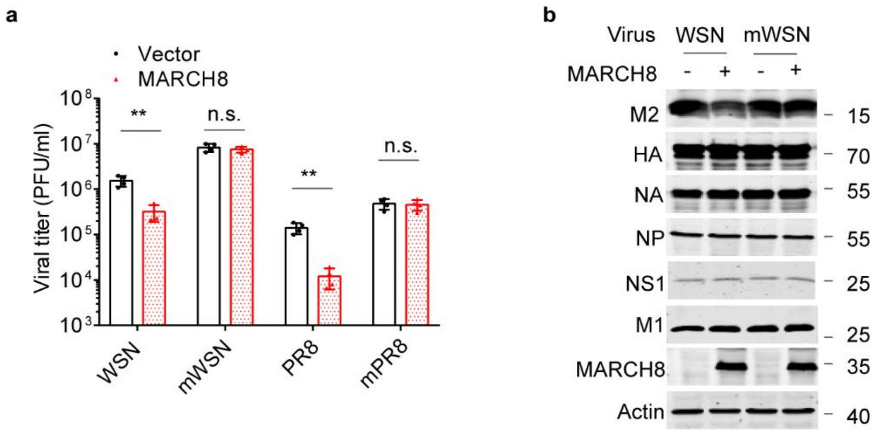

Supplementary Figure 6. Replication of WT and the K78R M2 IAVs in vitro. a. Control or MARCH8 over-expressing A549 cells were infected with WSN, PR8, recombinant WSN virus with K78R M2 mutant (m WSN) or recombinant PR8 virus with K78R M2 mutant (m PR8) (MOI = 0.1) for 24 h. The culture supernatants were collected and IAV titres were determined by plaque assay. Data shown are the means ± SD (n =3). **, P< 0.001; n.s., nonsignificant, unpaired two-tailed Student t-test. **b.** Control (-) or MARCH8-expressing (+) A549 cells were infected with WSN or mWSN virus (MOI = 0.2) for 8 h. Viral proteins in the cell lysates were examined by Western blotting.

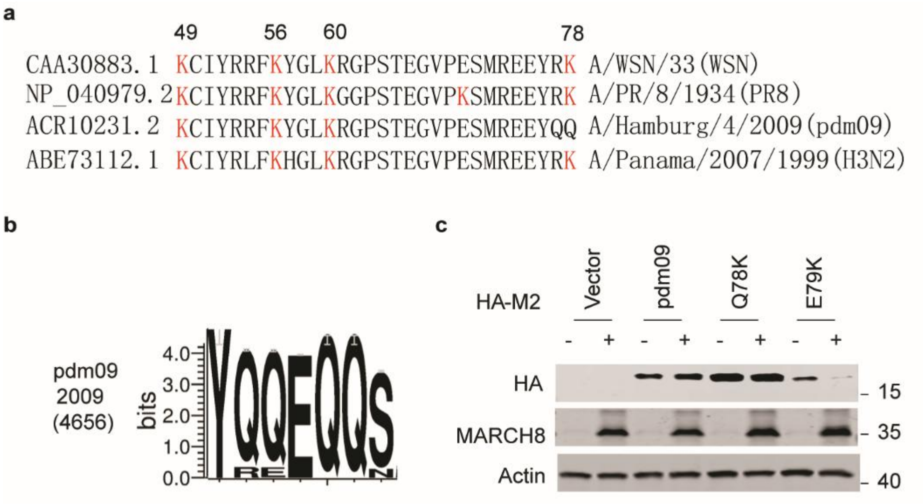

Supplementary Figure 7. MARCH8 mediated E79K mutant pdm09 M2 degradation. a. The cytosolic tail 49-78 amino acid sequences of M2 proteins from different IAV strains. The lysine residues are marked as red. **b.** Amino acid sequence logo of 4656 human pdm09 virus (H1N1) M2 proteins (residues 76-82). **c.** Effect of MARCH8 on the expression of pdm09 M2 mutants. The HEK293T cells were transfected with pdm09 M2 mutants and Vector or MARCH8. M2 and MARCH8 were detected with specific antibodies.

Supplementary Table 1

Characterization of PR8 WT and M2 K78Rmut viruses in vivo

Virus	MLD50 (PFU)	Dose (PFU)	% survival (no. of survivors/total no. tested)
WT	204.1	20	100 (5/5)
		50	80 (4/5)
		100	80 (4/5)
		200	60 (3/5)
		400	40 (2/5)
		800	0 (0/5)
		1600	0 (0/5)
K78R	47.7	20	100 (5/5)
		50	40 (2/5)
		100	20(1/5)
		200	0 (0/5)
		400	0 (0/5)
		800	0 (0/5)
		1600	0 (0/5)

Supplementary Table 2 Primers for qPCR

Gene	Forward primer (5'-3')	Reverse primer (5'-3')
IAV *NP* vRNA	GGCCGTCATGGTGGCGAAT	CTCAATATGAGTGCAGACCG TGCT
β-Actin	ACCAACTGGGACGACATGGAGA AA	TAGCACAGCCTGGATAGCAA CGTA
